# Comparisons of Two Different Treatment Methods for Impacted Maxillary Canines: A Retrospective Study

**DOI:** 10.3390/jcm13082374

**Published:** 2024-04-19

**Authors:** Anita Fekonja

**Affiliations:** 1 Department of Orthodontic Health Centre Maribor, Ulica Talcev 9, 2000 Maribor, Slovenia; anita-fekonja1@guest.arnes.si; 2 Faculty of Medicine, University of Maribor, Taborska ulica 8, 2000 Maribor, Slovenia

**Keywords:** impacted teeth, diagnosis, orthodontic appliance, orthodontic wire

## Abstract

**Background:** The impaction of the maxillary canine is a common occurrence, and orthodontists must be prepared to manage it. The purpose of this article is to present a study of the efficacy of a double-wire orthodontic appliance compared to a control group in the alignment of impacted maxillary canines in the dental arch. **Methods:** To diagnose an impacted maxillary canine, a panoramic radiograph was taken and a detailed analysis was performed by the same orthodontist. This article presents the results of 28 impacted maxillary canines with inclusion criteria of A2 (tooth angulation to the midline 16°–45°), V1 (vertical height of the tooth crown above the cementoenamel junction but less than half the length of the root of the maxillary lateral incisor), and O3 (medial position of the canine crown of more than half but less than the entire root width of the lateral incisor) positions in 21 patients (7 males and 14 females) with a mean age of 14.02 years (SD = 1.61) who were treated by the same orthodontist for impacted maxillary canines using a fixed double-wire orthodontic appliance. The analyzed data were compared with the control group (treated with a fixed orthodontic appliance and active ligature), which was matched for pretreatment age and the A2, V1, and O3 positions of the impacted maxillary canine. **Results:** With both methods, the impacted maxillary canines were aligned and guided into a correct position in the dental arch, but the mean active orthodontic traction of 31 ± 4.2 weeks in the SG was statistically significantly shorter (*p* < 0.05) in comparison to 37 ± 6.3 weeks in the CG, and the number of visits was statistically significantly (*p* < 0.05) fewer in the SG (5 ± 2) than in the CG (11 ± 5). **Conclusions:** The findings of this study are important to the field of orthodontics and show that the alignment of impacted maxillary canines with A2, V1, and O3 positions can be achieved with both methods, but with the double-wire appliance, the treatment time was shorter and the number of visits was statistically significantly fewer. The results of this study suggest that this approach may be more efficient and cost-effective compared to conventional methods.

## 1. Introduction

Impacted maxillary canines are a common finding in orthodontically treated patients, and their treatment is challenging due to their impact on the smile, facial esthetics, and function. The maxillary canines support the upper lip and alar base, which have an esthetic impact on the appearance of the face. The correct size, shape, and alignment of the maxillary canines play a role in the beauty of the smile. In addition, the maxillary canines also have important functional and gnathological significance: these teeth provide disocclusion of the posterior teeth during excursive movements [1]. A tooth can be defined as impacted if it remains infrabony after the completion of its normal development pattern [2,3]. After the third molars, maxillary canines are the most frequently impacted teeth [4,5,6,7,8,9], followed by the second premolars and maxillary central incisors [9]. The prevalence of impacted maxillary canines has been reported to be in the range of 0.8–4.7% [4,5,7,8,9], and is twice as high in females as in males [7,9]. Two-thirds of impacted maxillary canines are localized palatally, while only one-third involve buccal impaction [10].

The etiology of buccal impaction is primarily due to a lack of space in the dental arch [3]. Two theories have been proposed to explain palatal impaction: the genetic theory and the guidance theory. According to the genetic theory, the eruption anomaly of the maxillary canine is the result of a developmental disturbance of the dental lamina and may occur with other dental anomalies such as enamel hypoplasia, microdontia of the maxillary lateral incisor, and hypodontia of the second premolar. Evidence for this theory can be found in familial and bilateral occurrences, as well as in gender differences [11,12,13]. The guidance theory explains that the root of the maxillary lateral incisors serves as a guide for the eruption of the maxillary canines, in which they slide along their roots during an eruption. Thus, any interference with the guided eruption might result in a palatal impaction. Therefore, any disruption to this guided eruption can lead to palatal impaction. Disorders include hypodontia of the maxillary lateral incisor, supernumerary teeth, odontomas, displacement (transposition) of the tooth bud, and cystic or neoplastic formation [3,10,11,12,13,14,15,16].

It is important to diagnose impacted maxillary canines early in order to minimize the treatment complexity, cost, and time [17]. An accurate diagnosis of the impacted maxillary canines can be made by clinical examination (palpation and visual inspection) and radiographic examination [2,3,18]. Several clinical signs of impaction have been documented in the dental literature: prolonged retention of deciduous canines, delayed eruption of the permanent canine, absence of a labial canine bulge, presence of palatal bulge, distal tipping, or abnormal migration of the lateral incisors [13,18,19]; therefore, it is important to determine the normal eruption timing of teeth in the examined population [20]. Early diagnosis is important, and interceptive orthodontic treatment, such as space creation, may allow spontaneous eruption or an improvement in the position of the impacted teeth [3,8,21,22]. It is important to understand the predictive measures of impaction assessed using a panoramic radiograph: angulation and the mesial and vertical position of the impacted maxillary canine [23]. Cone beam computed tomography (CBCT) is a more accurate method than a panoramic radiograph to determine the position of the impacted maxillary canine in three dimensions and to analyze the possible presence of root resorption of the adjacent teeth. Furthermore, with CBCT, it is possible to precisely analyze the amount of bone around the impacted maxillary canines (IMCs) and the condition of the adjacent teeth [24].

The treatment of impacted maxillary canines usually requires an interdisciplinary approach. The treatment options are surgical exposure with orthodontic traction to align the malpositioned tooth, autotransplantation of the impacted canine, or extraction of the impacted maxillary canine and prosthetic treatment [25,26]. The usual treatment consists of appropriate surgical exposure of the impacted maxillary canines and complex orthodontic mechanics to bring them into an appropriate position in the dental arch [1,2,27,28]. Several surgical techniques for exposing impacted maxillary canines have been described in the literature. These techniques are classified into open and closed procedures and are associated with certain risks. Displaced canines may become ankylosed, lose their vitality, or cause problems for the adjacent teeth, such as root resorption, loss of vitality, or loss of hard or soft tissue. Insufficient gingival attachment is often the result of treatment [29,30]. Traction techniques accompanied by surgical exposure include conventional golden chain [1], extrusion spring [31], ballista spring [32], and cantilever on segmented arch techniques [33]. To prevent resorption, low forces should be applied during orthodontic treatment. The optimal force applied during orthodontic traction should be sufficient to move the tooth without damaging the tissue. The optimum force level is 0.3 to 0.4 N, while continuous and constant forces are important for the maximum biological response and minimal tissue damage [34]. Excessive forces can lead to the destruction of the periodontal tissue and resorption of the root [29]. Autotransplantation is a valuable alternative when surgical exposure and subsequent orthodontic treatment are difficult or impossible due to the unfavorable position of the impacted maxillary canine or if the patient refuses long-term orthodontic treatment [35].

The aim of this article is to present the use of a combined orthodontic wire for active orthodontic traction to bring the impacted maxillary canines with A2 (tooth angulation to the midline 16°–45°), V1 (vertical height of the tooth crown above the cementoenamel junction but less than half the length of the root of the maxillary lateral incisor), and O3 (medial position of the canine crown of more than half but less than the entire root width of the lateral incisor) positions into the dental arch. To evaluate the efficiency of active orthodontic traction with the combined orthodontic wire, we compared it with a control group treated with active ligature. We analyzed the time needed for active traction of the impacted maxillary canines into the dental arch and the number of required visits.

## 2. Materials and Methods

This study was reviewed and approved by the Institutional Review Board at the University Clinical Centre Maribor, Slovenia (03/14), and was conducted in accordance with the declaration of Helsinki in the Orthodontic Department of the Healthcare Centre Maribor. Informed consent was obtained from each subject or their parents.

**Figure 1:**
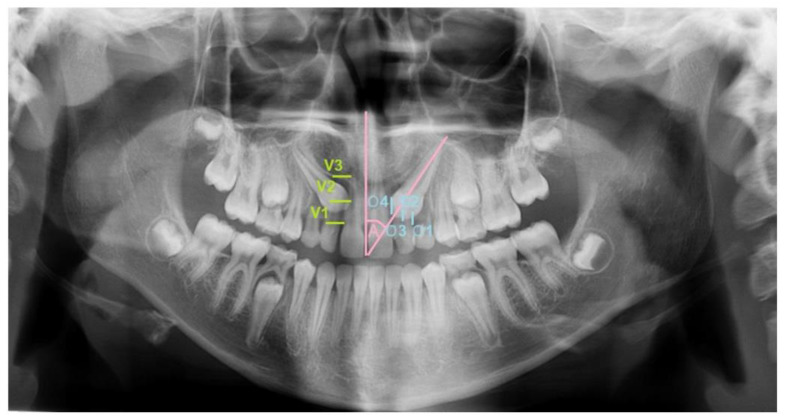
Angular and linear measurements of maxillary canine impaction on panoramic radiograph. Angulation of the maxillary canine to the midline (A; pink): The angle between the midline of the maxilla (sutura intermaxillaris) and the long axis of the impacted maxillary canine, which was divided into the following groups: Grade 1 (A1): 0°–15°. Grade 2 (A2): 16°–45°. Grade 3 (A3): ≥46°. Vertical height of the impacted maxillary canine (V; green): The crown height was graded in accordance with the adjacent maxillary lateral incisor: Grade 1 (V1): Above the cement–enamel junction (CEJ; blue), but less than half the length of the root of the maxillary lateral incisor. Grade 2 (V2): Between half the length of the maxillary lateral incisor root and its full length. Grade 3 (V3): More than the full length of the maxillary lateral incisor root. Overlap of the canine crown of the lateral incisor (O): The contact relationship between the cusp of IMC and the root of the adjacent maxillary lateral incisors: Grade 1 (O1): No horizontal overlap seen. Grade 2 (O2): Less than half the root width of the lateral incisor. Grade 3 (O3): More than half but less than the entire root width of the lateral incisor. Grade 4 (O4): Complete overlap of the root width of the lateral incisor or more.

A clinical examination and panoramic radiographs were performed to diagnose impacted maxillary canines. The material for the present study included the records of 159 patients with 205 impacted maxillary canines treated at the Orthodontic Department by the same orthodontist (AF) between 2002 and 2022. The inclusion criteria for this study were impacted maxillary canines with A2 (tooth angulation to the midline 16°–45°), V1 (vertical height of the tooth crown above the cementoenamel junction but less than half the length of the root of the maxillary lateral incisor), and O3 (medial position of the canine crown of more than half but less than the entire root width of the lateral incisor) positions identified by analyzing pretreatment panoramic radiographs (Figure 1). On the panoramic radiographs, no signs of root resorption or impairment of vitality of the lateral incisors were observed. Oral hygiene was fair.

The patients had no associated syndrome, alveolar cleft and/or palate, or previous tooth loss due to trauma, caries, periodontal disease, or orthodontic extraction. Gender and age were also recorded.

All panoramic radiographs were taken with the same equipment (Planmeca Promax, Helsinki, Finland) by an experienced dental radiology engineer before and after orthodontic treatment. The definition of the linear and angular measurements used (clinically and for treatment importance) are described and illustrated in Figure 1. The linear and angular measurements were measured to the nearest 0.5 mm and 1 degree, respectively.

All treated patients with impacted maxillary canines were divided into two groups: the study group (SG), in which the impacted maxillary canines were traced with a 0.013 CuNiTi wire, and the control group (CG), in which the impacted maxillary canines were traced with a 0.012-inch stainless steel ligature.

**Figure 2:**
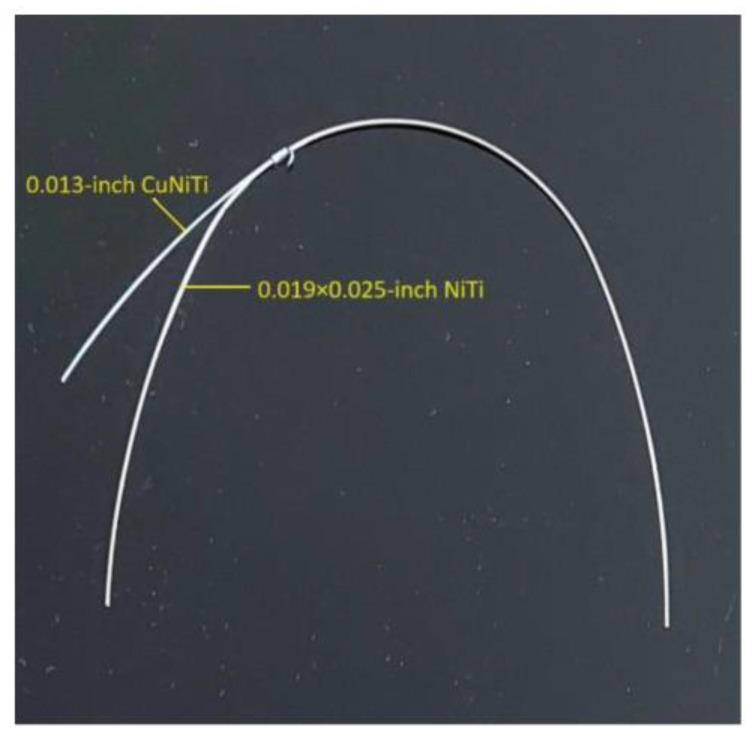
Double wire.

**Figure 3:**
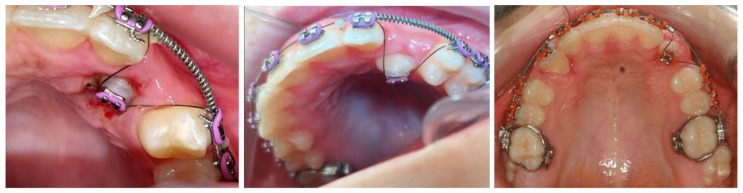
Double wire: the 0.019 × 0.025-inch NiTi wire was inserted into the brackets on the aligned maxillary teeth to stabilize them, and the 0.013-inch CuNiTi archwire was fixed with an elastic ligature in the bracket on the impacted maxillary canine to track it into the dental arch.

The double orthodontic wire consists of an additional 0.013-inch CuNiTi wire connected to the basal 0.019 × 0.025-inch NiTi wire (Figure 2). The 0.019 × 0.025-inch NiTi wire is the part that stabilizes the teeth in the upper dental arch after alignment, and the 0.013-inch CuNiTi superelastic wire is part of the active traction to move the maxillary impacted canines into the dental arch (Figure 2 and Figure 3).

All patients were willing to undergo orthodontic–surgical treatment, although they were informed about the duration of treatment and the risks of therapy.

A 0.022 × 0.028-inch Roth appliance was bonded to the existing teeth by the same orthodontist, and light continuous wires of 0.012-inch NiTi, 0.016-inch NiTi, 0.018-inch NiTi, 0.16 × 0.22-inch NiTi, and, finally, 0.019 × 0.025-inch NiTi were inserted. In all patients, sufficient space was created for the impacted maxillary canine by an open coil spring.

The crown of the impacted maxillary canine was surgically exposed by a maxillofacial surgeon under local anesthesia when the space for the impacted maxillary canine was created. Based on the radiographic analysis of the impacted maxillary canine position, in all patients, an open technique was recommended. After bonding the bracket to the IMC, two different treatment methods were used: double wire (SG) or active 0.012-inch-long stainless steel ligature (CG).

In the SG, the bonded bracket was attached to the impacted maxillary canines with the ligature on the 0.013-inch CuNiTi orthodontic wire. The other part of the combined wire, the 0.019 × 0.025-inch NiTi wire, was inserted into the other upper brackets (Figure 3) to stabilize the teeth in the upper arch. The effect produced by the 0.013-inch CuNiTi wire is a summary of the properties of the wire itself and the geometric forces, and it actively tracks the impacted maxillary canine into its final position.

**Figure 4:**
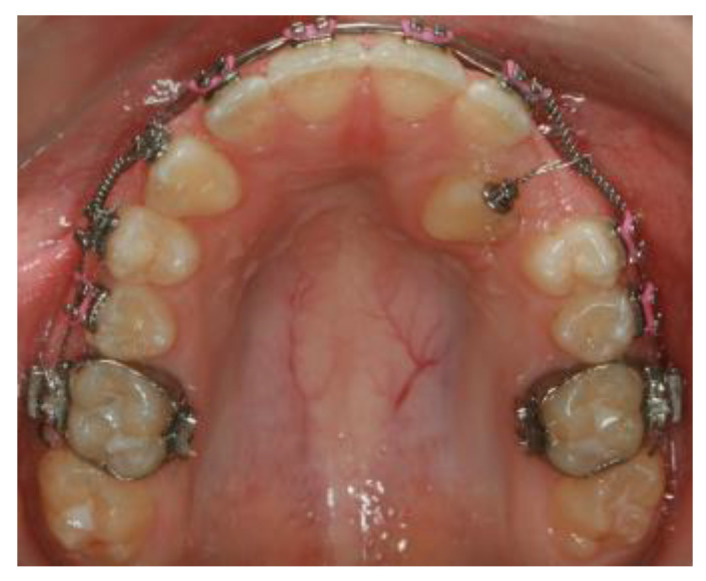
The 0.019 × 0.025-inch NiTi wire was inserted into the brackets on the aligned maxillary teeth to stabilize them, and the long stainless steel ligature was attached to the impacted maxillary canine with a bonded bracket.

In the CG, the bonded bracket on the impacted maxillary canine was attached to the 0.019 × 0.025-inch NiTi wire inserted into the other upper brackets with the long stainless steel ligature wire. This long ligature was activated approximately every three weeks to track the impacted maxillary canine into the dental arch (Figure 4).

These two methods have side effects such as locations for plaque accumulation and soft tissue irritation.

The duration of active orthodontic traction to bring the impacted maxillary canines into the dental arch was recorded by the treating orthodontist (AF). The start of the orthodontic traction of the impacted maxillary canine was defined as the time that elapsed between the surgical exposure technique (after leveling the teeth and creating space for the impacted maxillary canine) and the time when the impacted maxillary canine was in its correct position in the dental arch. The duration of active orthodontic traction was measured in weeks. From this point of view, additional anomalies in the dental arch (e.g., misaligned teeth) before surgical exposure and the time to establish maximum intercuspation (e.g., intermaxillary elastics) after the impacted canine was in the correct position in the dental arch had no influence on the duration of active orthodontic traction. The purpose of this study was to compare the time of active traction between the two methods.

The number of visits required for active orthodontic traction was also recorded. A post-treatment panoramic radiograph was performed on each patient to evaluate the proper (parallel) position of the traced canines and any damage to the roots of the adjacent teeth.

### Statistical Analysis

Intra-examiner reliability was evaluated by re-measuring twenty radiographs two weeks after the first measurement by the same examiner (AF). Error analysis was performed using a paired *t*-test.

The data were collected and entered into the spreadsheet program Microsoft Office Excel 19. The distribution of the numerical variables (age of subjects, duration of active treatment, number of visits) was presented with the corresponding values: mean and standard deviation. The Pearson rank test was used to determine the independence between categorical variables.

An independent *t*-test at a significance level of <0.05 was used to assess the differences in age between the groups. We analyzed the difference in active treatment time and number of visits between the groups using a chi-square test with a *p*-value of <0.05 as a standard for a statistically significant difference.

The sample size was calculated using G-Power version 3.0.10 (Universitate Keil, Keil, Germany). The calculated minimal sample size was 24.6. Therefore, we decided that 28 impacted maxillary canines in the study group and 26 impacted maxillary canines in the control group should be a sufficient sample size for this study.

## 3. Results

A total of 205 impacted maxillary canines were observed in the patients treated by the same orthodontist at the Orthodontic Department between 2002 and 2022. Of the 205 impacted maxillary canines, a total of 54 met the inclusion criteria of A2 (tooth angulation to the midline 16°–45°), V1 (vertical height of the tooth crown above the cementoenamel junction but less than half the length of the root of the maxillary lateral incisor), and O3 (medial position of the canine crown of more than half but less than the entire root width of the lateral incisor) positions.

We analyzed the results of the 28 impacted maxillary canines in 21 patients (7 males and 14 females) with a mean age of 14.02 years (standard deviation 1.61 years) treated with a double-wire appliance (SG), and those of a control group (CG) involving 26 IMCs in 20 patients (6 males and 14 females) with a mean age of 13.88 years (standard deviation 1.58 years) treated with active ligation. There were no statistically significant differences (*p* > 0.05) in age and gender between the two groups.

We found that the mean active orthodontic traction of 31 ± 4.2 weeks in the SG was statistically significantly shorter (*p* < 0.05) in comparison to 37 ± 6.3 weeks in the CG (OR = 4.01; 95% CI, 1.31–8.15), and that there were statistically significantly (*p* < 0.05) fewer visits in the SG than in the CG (OR = 4.01; 95% CI, 1.04–9.75) (Table 1).

No signs of root resorption, impairment of vitality, or other damage to the lateral incisors and other teeth were observed during treatment and on post-treatment panoramic radiographs. At the end of the treatment, periodontal assessment showed healthy marginal tissue.

## 4. Discussion

Impacted maxillary canines are not uncommon in orthodontic practice, and the treatment of this condition is usually a challenge for the orthodontist. It has been found that the prevalence of impacted maxillary canines is twice as high in females [7,8,9]. In this study, the incidence was also found to be higher in females than in males.

The treatment of impacted maxillary canines plays an important role in the good function of the teeth and the esthetic appearance of the smile [1,27,28]. This includes combined orthodontic and surgical treatment, which is essential to determine the correct diagnosis, prognosis, course, and outcome of treatment. An early diagnosis of impacted maxillary canines is important to reduce the complexity, duration, and cost of the treatment [3,8,21]. The clinical examination of patients up to the age of nine years is advisable to assess the displacement of the canines from their normal position. Important clinical signs of impacted maxillary canines are the persistence of the corresponding deciduous canine when the contralateral permanent canine has already erupted, and the absence of a labial bulge 1 to 1.5 years before the expected time of tooth eruption [36]. Another sign of an eruption disorder is a deviation from the normal eruption sequence, e.g., when the second molar erupts before the canine [37]. The shorter roots of the maxillary lateral incisor, as reported by Kucukkaraca [38], and hypodontia of the maxillary lateral incisor, as reported by Baccetti [14] and Moyers [16], may be potential factors for the impaction of the maxillary canine, which should be considered during clinical examinations and in the analysis of panoramic radiographs. Warford et al. [39] reported a greater possibility of impaction when the canine overlaps the long axis of the lateral incisor. The diagnosis of an impacted maxillary canine is confirmed through a clinical examination and a radiographic evaluation and is crucial for planning treatment. Untreated impacted maxillary canines can cause serious problems [2,3,18]. In our study, the diagnosis was made by both clinical examinations (inspection, palpation) and panoramic radiography.

Two main surgical techniques are recommended for exposing impacted maxillary canines: open and closed [28]. In this study, the open technique was used to remove the tissue over the impacted maxillary canines. The main advantage of the open technique is the short surgical duration, and the main disadvantage is the prolonged postoperative recovery, sensitivity, and plaque accumulation [40]. The selection of an appropriate conservative surgical technique with adequate orthodontic forces plays a very important role in the success of the treatment.

The aim of orthodontic treatment in impacted maxillary canines is to bring the teeth into the correct position by applying forces to them. The ideal force is one that causes tooth movement without damaging the teeth or periodontal tissues. The factors that determine the periodontal outcome of IMCs are mainly the quality of the periodontium, the location of the eruption point, and the force applied to it [35]. In recent decades, material science has made rapid progress. In the field of orthodontics, not only have materials improved, but philosophies have also changed. Orthodontic wires, which generate the biomechanical force for impacted maxillary canine movement through brackets, are central to the practice of the profession [41,42]. Metals, polymers, and composite materials are used for the manufacture of orthodontic wires. Each material has its advantages, and the clinician must have a good knowledge of the mechanical and physical properties of the wires to determine their clinical applicability and achieve a satisfactory and predictable result [43]. The main advantage of using NiTi archwires in orthodontics is the release of continuous and controlled forces that produce the desired tooth movement under elastic deformation. This efficient tooth movement in a short period of time under large deflections is important during the alignment phase of orthodontic therapy to shorten the treatment time and achieve a positive biological response by avoiding the root resorption process. CuNiTi represents the next generation of superelastic and shape memory wires as they reduce hysteresis and allow for a precise transformation temperature [42,43]. Compared to conventional NiTi wires, CuNiTi wires develop about 20% less load force. The decrease in the force generated by CuNiTi is less than that of NiTi alloys. This explains the clinical efficiency of CuNiTi when the teeth continue to work close to their intended position [40]. Stainless steel wires generate higher forces acting over shorter time periods because they have a lower springback capacity and also store less energy than beta-titanium or nickel–titanium wires [44].

Several methods of delivering physiologic force for traction in maxillary impacted teeth were presented. Apart from the conventional golden chain and elastic approaches, there are also other auxiliary mechanics that allow the traction of an impacted maxillary canine into the dental arch. Conventional alignment may put the adjacent lateral incisors at risk of resorption due to the pronounced torque from the wire and from guiding the incisor close to the resorptive follicle of the impacted canine [45].

It is important to control the movement of impacted maxillary canines to prevent the root resorptions of lateral incisors. This can be allowed by the temporary anchorage device reported by Koscis et al. [46]. Singh et al. [32] reported another advantageous method that can be used before and during the leveling phase. Nakandakari et al. [33] reported an efficient and predictable outcome of using the segmented arch technique due to its static mechanics. In this study, in the first phase, sufficient space was created and then the impacted canine was traced into the correct position in the arch with two different methods.

Backer et al. [47] reported that if the apex of the impacted maxillary canine is in the line of the arch in the buccopalatal plane and the mesiodistal plane, the crown of the impacted tooth will only need to be tipped into the correct position in the dental arch with a relatively simple biomechanical exercise. In this study, the inclusion criteria for the position of the impacted maxillary canine (angulation, tipping, vertical direction) allowed for the same biomechanic.

As reported by Vasoglou et al. [34], low and continuous force is important for maximum biological response and to prevent tissue damage. In this study, continuous force was enabled by the double wire. Compared to the double arch, the force during stainless steel ligature activation is less continuous, which is similar to the elastic chain technique. Chains undergo permanent deformation and force decay over time. In addition, chains are suitable for microorganism proliferation.

We believe that the duration of active orthodontic traction is also important. A longer duration of treatment can be associated with tissue damage (demineralization, caries, gingivitis) due to plaque accumulation. During pandemics (e.g., COVID-19), the availability of an orthodontist and, thus, the frequency of visits to the orthodontist are also important factors. In this study, we compared the time of active orthodontic traction and the number of visits required to trace an impacted maxillary canine into the dental arch according to the use of two different methods (CuNiTi archwire and stainless steel).

Warford et al. [39] and Aras et al. [48] reported that impacted canines with a greater angle between the midline of the maxilla (sutura intermaxillaris) and the long axis of the impacted maxillary canine, a higher vertical position of the tooth crown above the cement–enamel junction, and a more mesial position of the crown of the canine have a poorer treatment prognosis. Crescini et al. [49] reported that the active treatment time in patients with impacted maxillary canines is proportional to the overlap of the lateral incisors and the angle between the long axis of the impacted tooth and the midline. The researchers reported that the treatment time increased by one week for every 5 degrees of angle increase. In this study, 54 impacted maxillary canines met the inclusion criteria of A2 (the tooth angulation to the midline 16°–45°), V1 (the vertical height of the crown above the cement–enamel junction but less than half the length of the root of the maxillary lateral incisor), and O3 (medial position of the canine crown of more than half but less than the entire root width of the lateral incisor) positions. With both methods, the impacted maxillary canines were aligned into the dental arch, but the mean time of active orthodontic traction was shorter (31 ± 4.2 vs. 37 ± 6.3) and the number of visits was statistically significantly fewer (5 ± 2 vs. 11 ± 5) in the study group compared to the control group. In the control group, a long stainless steel ligature was activated approximately every three weeks to track the impacted maxillary canines in the dental arch. The force is strong upon activation and then gradually decreases. In the study group, the force of the double wire was more continuous and constant, so the patients did not need as many visits. The results are not comparable, as there are no similar inclusion criteria (position of impacted maxillary canines) in other research in the literature.

Our study has some limitations. The study was limited to Caucasian orthodontic patients. Moreover, there was a relatively low number of subjects included in this study due to the low number of patients who met the inclusion criteria in terms of the position of the impacted maxillary canines. Although this study may not be representative of the population as a whole, the result is beneficial to orthodontists, as it provides a method for the faster active traction of impacted maxillary canines with fewer visits (clinical controls). Treatment with fewer clinical visits is particularly important during pandemics (such as COVID-19) when visits to the orthodontic clinic are limited. Another limitation of this study is the analysis of the impacted maxillary canine position on a panoramic radiograph, as not all patients treated between 2002 and 2015 had a CBCT. In our clinical practice, we only started using CBCT after 2015. However, the increased cost and radiation exposure associated with using CBCT limit its routine use.

## 5. Conclusions

The findings of this study are important to the field of orthodontics and show that the alignment of impacted maxillary canines with A2 (tooth angulation to the midline 16°–45°), V1 (vertical height of the tooth crown above the cementoenamel junction but less than half the length of the root of the maxillary lateral incisor), and O3 (medial position of the canine crown of more than half but less than the entire root width of the lateral incisor) positions can be achieved with both methods, but with the double-wire appliance, the treatment time was shorter and the number of visits was statistically significantly fewer. The results of this study suggest that this approach may be more efficient and cost-effective compared to conventional methods.

## Figures and Tables

**Table 1:** Analyzed patients with included values: A2, V1, and O3.

	Gender (*n*)	IMC (*n*)	Age Mean ± SD (Years)	Active Orthodontic Traction Mean ± SD (Week)	Visits Mean ± SD (*n*)
Study group	Male; 7 Female; 14	28	14.02 ± 1.61	31 ± 4.2	5 ± 2
Control group	Male; 6 Female; 14	26	13.88 ± 1.58	37 ± 6.3	11 ± 5

SD, standard deviation; IMC, impacted maxillary canine; A2, angulation of the maxillary canine to the midline (16°–45°); V1, vertical height of the impacted maxillary canine (above the cementoenamel junction but less than half the length of the maxillary lateral incisor root); O3, overlap of the canine crown of the lateral incisor (more than half but less than the entire root width of the maxillary lateral incisor).

## Data Availability

The data presented in this study are available on request from the corresponding author.

## Abbreviations

Impacted maxillary canine	IMC
Cone beam computed tomography	CBCT
Nickel–Titanium archwire	NiTi
Copper–Nickel–Titanium archwire	CuNiTi
